# ASO Author Reflections: From BRPC to LAPC—Rethinking Resectability in Pancreatic Cancer

**DOI:** 10.1245/s10434-025-17676-0

**Published:** 2025-06-19

**Authors:** Jacob Ghotbi, Ingvild Farnes, Knut Jørgen Labori

**Affiliations:** Department of Hepato-Pancreato-Biliary (HPB) Surgery, Oslo University Hospital, Institute of Clinical Medicine, University of Oslo, Oslo, Norway

## Past

We have previously demonstrated that, in a population based, intention to treat cohort of patients with borderline resectable (BRPC) and locally advanced pancreatic cancer (LAPC), the resection rates are low (47% and 13%, respectively), but survival outcomes remain comparable to patients with primary resectable disease who undergo surgery, justifying the role of pursuing surgery for these cases.^1^

## Present

In the current analysis of the NORPACT-2 trial, we examined outcomes in anatomical subgroups of patients with BRPC and LAPC, identifying how different degrees of vascular involvement affect resectability and survival outcomes.^2^ Contrasting the National Comprehensive Cancer Network (NCCN)^3^ with the Dutch classification system,^4^ we revealed notable discrepancies. First, patients categorized as BRPC by NCCN but LAPC under the Dutch system—due to more extensive vascular involvement—had particularly poor outcomes and low resection rates. While the NCCN system was predictive of resectability, it did not correlate with survival outcomes. In contrast, the Dutch system, by shifting more advanced cases from BRPC to LAPC, predicted both the likelihood of surgical resection and survival. Additionally, applying the Louisville system^5^ to our LAPC cohort identified anatomical subgroups with a meaningful likelihood of proceeding to surgery, challenging the traditional view of LAPC as uniformly unresectable.

## Future

The ongoing NORPACT-3 trial (2025–2028) aims to build on these findings by prospectively enrolling BRPC and LAPC patients from all hepato-pancreato-biliary (HPB) centers in Norway. The goal is to develop a response-adaptive, personalized treatment algorithm to enhance resection rates and survival. Key elements include integrating dynamic response monitoring (e.g., CA19-9, CEA, circulating tumor DNA), baseline molecular profiling (e.g., *KRAS*/MSI), and advanced imaging (positron emission tomography [PET]/computed tomography [CT]) at diagnosis and during treatment. The study also incorporates translational research, with analyses of serum metabolic and lipoprotein profiles and circulating tumor DNA to uncover predictive biomarkers and improve our understanding of tumor biology.
